# COVID-19 pandemic: ethical issues and recommendations for emergency triage

**DOI:** 10.3389/fpubh.2023.1160769

**Published:** 2023-05-05

**Authors:** Leqian Wu, Xiangjin Kong

**Affiliations:** College of Humanities and Social Sciences, Dalian Medical University, Dalian, Liaoning, China

**Keywords:** Coronavirus Disease 2019, triage inaccuracy, emergency department crowding, high-tech, safety, autonomy, resource waste, care ethics

## Abstract

The current epidemic of Coronavirus Disease 2019 (COVID-19) has become a public health event worldwide. Through ethical analysis of a series of epidemic prevention phenomena and epidemic prevention measures taken by the Chinese (and other countries) government and medical institutions during the COVID-19 pandemic, this paper discusses a series of ethical difficulties in hospital emergency triage caused by the COVID-19, including the autonomy limitation of patients and waste of epidemic prevention resources due to over-triage, the safety problem of patients because of inaccurate feedback information from intelligent epidemic prevention technology, and conflicts between individual interests of patients and public interests due to the “strict” implementation of the pandemic prevention and control system. In addition, we also discuss the solution path and strategy of these ethical issues from the perspective of system design and implementation based on the Care Ethics theory.

## 1. Introduction

Since the end of 2019, there has been a global outbreak of Coronavirus Disease 2019 (COVID-19). Cases of breakthrough infections have occurred due to the emergence of mutant strains of SARS-CoV-2, the virus that causes COVID-19, as a result of ongoing genetic mutations (1). SARS-CoV-2 is transmitted from person to person mainly through airborne droplets and direct contact (2, 3). COVID-19 is characterized by rapid spread, strong infectivity, and general susceptibility of the population, which can easily cause outbreaks and epidemics. The current Diagnosis and Treatment Plan for COVID-19 (Trial Version 9) issued by the National Health Commission of the People’s Republic of China, focuses on symptomatic management and respiratory support (4) and there exists a lack of effective antiviral treatment for COVID-19, making it an epidemic infectious disease that endangers the lives of people all over the world.

Before the outbreak of COVID-19, emergency departments (EDs) have been facing a series of challenges around the world, overcrowding, dramatic increases in patient volume, insufficient numbers of experienced medical staff, resulting in longer emergency wait times and inaccurate triage, negatively impacting the overall operational efficiency of emergency departments (EDs), increased patient safety risks and decreased satisfaction, and staff satisfaction burnout (5). The epidemic of COVID-19 has exacerbated this set of problems and posed new ethical challenges. Similarly, this greatly highlights the shortcomings of emergency departments in dealing with public health events that may seriously affect a wide range of people. (6). During the epidemic, emergency pre-examination and triage changes in terms of location arrangements and patient service flow. In addition, screening and triage focus on preventing the spread of as many potential sources of infection as possible (7). For numerous general hospitals, the emergence of COVID-19 epidemics has brought the original triage system and process into conflict with epidemic prevention and control measures. Specifically, the inaccuracy of triage, overcrowding in emergency departments (EDs), and the use of epidemic prevention measures, like intelligent high-tech (including face recognition access control system with temperature measurement, tele-triage technology) contribute to a series of ethical issues, such as issues of safety guarantee, privacy protection, or autonomy guarantee of patients and issues of rational use of medical resources. These ethical issues are worthy of in-depth discussion and analysis.

## 2. New challenges to hospital emergency triage brought by the COVID-19 epidemic

### 2.1. Inaccuracy of emergency triage: over-triage and under-triage

There are numerous emergency triage scales that are commonly utilized, among which the Emergency Severity Index (ESI) scale was developed in the United States (U.S.) and is now used by an increasing number of EDs worldwide (8). During the COVID-19 in South Korea, Park JH et al. improved and implemented a fast and flexible screening system (used patient medical history and radiological testing to screen visiting patients, and created a separate isolation area for the investigated patients) (9). In China, in order to avoid nosocomial infections, all medical institutions have issued official announcements requiring patients entering the emergency department to have a 48-h negative nucleic acid test before being allowed to enter the emergency department. This proactive identification isolation strategy was effectively applied in the COVID-19. However, insufficient attention is paid to the triage of infectious diseases in most triage systems currently applied.

Moreover, as the spread of the epidemic continues to change, the timeliness and proximity of the triage system is also insufficient. During the epidemic, triage nurses need to not only classify patients with critically ill patients but also perform epidemiologic screening and screen for patients infected with COVID-19. Currently, real-time reverse transcription–polymerase chain reaction (rRT-PCR) is considered the COVID-19 standard for most diagnostic studies, but there are data suggesting that the sensitivity of a single test ranges from 60 to 78% (10). Clinical symptoms of COVID-19 are extremely similar to influenza, including fever, tiredness, dry cough, and other respiratory symptoms, occasionally accompanied by nasal congestion, runny nose, and sore throat. Except for fever and olfactory/gustatory disorders, the medical history and physical examination results do not help to distinguish COVID-19 from other infectious diseases (such as influenza) that mimic SARS CoV-2 (10). In addition, people can be infected with both influenza virus and SARS-CoV-2 and manifest symptoms of both influenza and COVID-19. However, the risk of transmission from asymptomatic or mildly symptomatic patients has not yet been fully quantified (11–13). Therefore, it is extraordinarily difficult to identify patients with COVID-19 infection.

The need for epidemic prevention and control has led to a “nursing shortage” in a lot of countries, so the hiring of “new nurses” has elevated to meet the growing and changing needs of the epidemic (14). However, these “new triage nurses” are inexperienced in emergency triage, resulting in inaccurate triage, either over-triage or under-triage. Under-triage refers to any patient assigned to a triage tier of lower acuity on arrival (nurse-assigned) than at the close of encounter (physician-assigned), while the over-triage is the opposite (8). In fact, if triage nurses only rely on a small amount of information such as symptoms, epidemiological history, and vital signs of patients, without the combination of valid laboratory and imaging findings, to make a triage, they are likely to confuse other infectious diseases with COVID-19, culminating in inaccurate triage. In addition, triage nurses may mistakenly triage patients without COVID-19 symptoms and in low-risk areas to the fever clinic or may be engaged in positive defensive medical [positive defensive medicine is specific to overutilization, excessive testing, over-diagnosing, and overtreatment (15)] behaviors to include all patients in the category of potential COVID-19 infection using their subjective judgment and mistakenly triage patients to the fever clinic, all of which belong to over-triage. On the contrary, triage nurses also may incorrectly triage patients with COVID-19 symptoms to the ED for visit or refuse to accept acute patients in moderate-or high-risk areas, patients with a contact history with suspected COVID-19, or patients with suspected COVID-19 due to passive defensive medical behavior [negative defensive medicine is specific to avoiding, referring, or transferring high-risk patients (14)], all of which are under-triage. Over-triage or under-triage can both raise a host of ethical issues.

### 2.2. Difficult-to-dredge ED: intensifying crowding

Hospital EDs worldwide are notoriously overcrowded. A key feature of overcrowding is that when demand exceeds capacity, the various parts of the system form queues, which leads to an extended average ED length of stay (ED LOS) (16). In fact, the number of ED visits may decrease at the beginning of the COVID-19 epidemic. A survey showed that U.S. ED visits dropped 23% for heart attacks, decreased 20% for strokes, and reduced 10% for diabetic emergencies between March and May 2020 (17). During the COVID-19 epidemic in Italy, the number of pediatric patients admitted to hospital for the Pediatric Emergency Department (pED) due to trauma decreased by 68.8% (18). This phenomenon may be mainly due to the fear of patients being placed in mandatory isolation because of fever and/or respiratory symptoms or the fear of possible COVID-19 infection, leading some mild patients not to go to the ED.

However, critically ill patients do not think so much and often choose to go to the ED as soon as possible or be transported by ambulance to the ED because of the urgency and suddenness of their disease. Although the number of ED visits reduces during the initial phase of the epidemic for all of these reasons, it does not mean that ED crowding is relieved because the ensuing epidemic prevention measures complicate the triage process, resulting in longer triage times for individual patients. Emergency crowding was not improved through the reduction in the number of visits due to the epidemic and may have even increased after the number of emergency patients resumed. In addition, in pEDs, children are exposed to a high frequency of triage errors because they involve asking their guardians for relevant information and often involve emergency classification of high-risk children in complex and stressful environments (19). If the pED is overcrowded, the triage error rate will be even higher. Therefore, whether it is adults or vulnerable groups of older adults and children, ED overcrowding can trigger delays in emergency care, increased probability of nosocomial infections, and even death of patients due to untimely rescue.

### 2.3. Application of high-tech for epidemic prevention: inaccurate assessment of patient information

Tele-medicine refers to a medical technology that uses electronic information and telecommunication technologies to achieve a remote clinical diagnosis, health education, and public health management (20). Tele-triage is a type of tele-medicine, which is majorly utilized for patients whose conditions are not immediately life-threatening but still require emergency medical treatment. In general, most medical services are provided face-to-face. Nevertheless, during the anti-epidemic period, large quantities of tele-medicine infrastructures have become frequently applied. Therefore, the epidemic of the COVID-19 has severely disrupted this face-to-face form of medical service delivery, thus pushing virtual medical treatment and triage technologies to the cusp. In response to the pandemic, a tele-triage process was implemented by the Department of Ophthalmology and Visual Sciences at the University of Illinois at Chicago, Illinois Eye and Ear Infirmary (21). Triage nurses can pre-assess the information of patients, including age, symptoms, and epidemiological history, via the Internet or telephone. However, accuracy is hard to be guaranteed because detailed assessments cannot be performed face-to-face. In addition, when patients are screened, it is difficult to predict whether the condition of patients can deteriorate during tele-triage or before seeking medical attention after triage. All of these are drawbacks of virtual triage devices.

Meanwhile, with the development of the epidemic and the prolongation of epidemic time, various social institutions (including government agencies at all levels, enterprises and institutions, etc.) are increasingly employing fever examination as a mechanism to measure whether the population, travelers, employees, and patients are infected with COVID-19 (22). In China, both community hospitals and large general hospitals are equipped with health code face recognition temperature measurement and access control all-in-one machines at the entrance of outpatient departments and EDs. This device is docked to the national government affairs platform and big data platforms in various places and thus can read and verify personal health code status, dynamic trip codes, nucleic acid test results, and vaccination information. Patients and their families can perform face recognition, document comparison, and infrared temperature measurement by swiping their ID cards, social security cards, and health codes, which can rapidly screen for people in medium-and high-risk areas. Although the widespread use of such devices has largely enhanced the efficiency of epidemic prevention and screening, there may still be potential inaccuracies, uncertainties, and limitations in their information feedback. In particular, significant measurement errors exist in infrared temperature measurement. For example, when the outdoor temperature is below zero, the information is usually inaccurate when entering the room and immediately measuring the body temperature through infrared light.

## 3. Ethical dilemma of emergency triage under the COVID-19 epidemic

The nursing ethical principles include autonomy, non-maleficence, beneficence, justice, fidelity, and veracity (23). Nurses must be patient-centered and conscientious in fulfilling the aforementioned nursing ethical principles in the course of nursing practice. The International Council of Nurses (ICN) launched its newly revised Code of Ethics for Nurses on October 20, 2021, which highlights that nurses should possess values of the profession, like respect, justice, empathy, responsiveness, caring, compassion, trustworthiness, and integrity (24).

**Figure 1:**
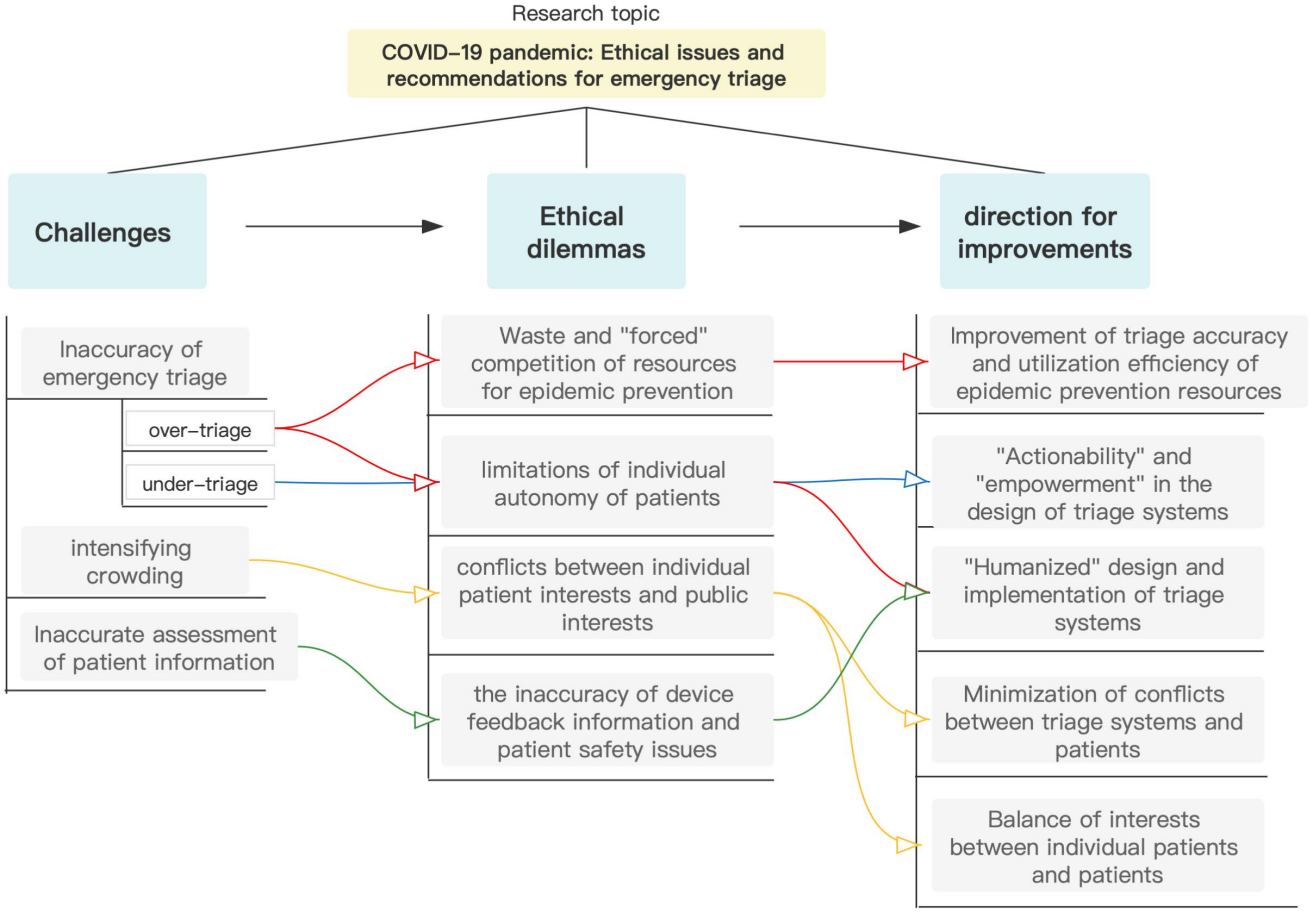
The relationship between challenges, ethical dilemmas, and directions for improvement in emergency triage.

Indeed, inaccuracies in triage, ED crowding, and inaccurate assessment of tele-triage information existed before the epidemic but were exacerbated by the COVID-19 epidemic. The COVID-19 epidemic constantly facilitates the informatization and intelligence of all medical means (like the use of face recognition temperature measurement and access control all-in-one machine born out of epidemic prevention needs) and also impacts the emergency triage system. All the medical institutions are forced to take such measures urgently because of the epidemic prevention work, while the possible ethical issues induced by these measures are generally ignored. It may also be difficult for nurses to practice nursing values because of multiple factors (environmental changes, work stress, fear of infection, responsibility to their families, increased workload, etc.). Figure 1 summarizes the challenges, ethical dilemmas, directions for improvement, and the relationship between them all in emergency triage during an outbreak.

### 3.1. Application of intelligent epidemic prevention technology: the inaccuracy of device feedback information and patient safety issues

The World Health Organization defines patient safety as the absence of preventable harm to patients and the prevention of unnecessary harm by health care professionals (25). In Groundwork of the Metaphysics of Morals, Kant suggests that only actions motivated by moral responsibility have the value of moral goodness: “to preserve one’s life is one’s duty, and besides everyone has an immediate inclination to do so.” (26). The right to life is the basis of all rights of patients. Nevertheless, under-triage, increased emergency crowding, and the uncertainty of high-tech can all threaten the lives of patients. Intelligent devices can inevitably lead to the ethical issue of privacy leakage, which is a cliché. This paper does not dwell on this issue but focuses on the ethical issues arising from the inaccuracy, uncertainty, and limitations of such devices.

Tele-triage (via the Internet or telephone) has emerged as a means of reducing ED crowding and cross-infection. The inaccuracy of tele-triage predisposes to delayed untimely treatment for acute patients. A Paris study reported that 1% of misdiagnosis of tele-triage in a primary and secondary ophthalmic emergency office contributed to delayed emergency care during COVID-19 lockdown (27). This survey data only reflects the conditions of the ophthalmic ED. Nonetheless, the rate of misdiagnosis may change as the target population expands. Due to the cross-cutting and difficult-to-identify symptoms of acute disease, like acute symptoms of abdominal pain that may suggest myocardial infarction, problems are likely to result from tele-triage in special populations. For instance, since the older adults are not sensitive to pain, an older adults patient with a sudden headache suffers not necessarily severe headache symptoms, but he may develop a subarachnoid hemorrhage. Therefore, is there a possibility that the subjective non-severe headache of the patient may mislead the triage nurse? Misdiagnosis can be largely caused by the subjective description of patients coupled with the subjective empirical judgment of triage nurses, resulting in the patient missing the optimal treatment time. Is it the most timely and effective if these patients go to the ED for treatment in the first instance without tele-triage?

Besides, face recognition temperature measurement devices still have the inaccuracy of temperature measurement although their extensive utilization does open a novel way to diminish the burden of epidemic prevention work. In Honduras, relevant research designs manifested 82% of accuracy and 14% of error for face recognition and temperature measurement devices (28). It has also been investigated that infrared temperature measurement using non-contact infrared thermometers underestimates body temperature, which may be insufficient to detect fever. There exist statistically significant correlations of infrared body temperature with outdoor temperature, relative humidity, and wind speed, which may trigger normal results in subjects who are truly febrile (29). That is, if an emergency patient is unaware of his low fever and visits the ED for non-febrile symptoms, the face recognition temperature measurement device may classify him in the range of normal body temperature when identifying and measuring his body temperature, leading him to enter the ED. In the ED, the triage nurse takes a second temperature measurement using an infrared temperature gun, which may still display a normal temperature, and subsequently this patient “successfully “completes the visit. Does this suggest that a loophole exists in the process? The inaccuracy of the device is likely to induce a major outbreak of nosocomial infection, threatening the safety of other emergency patients and even emergency staff.

### 3.2. The “strict” implementation of the epidemic prevention and control system: conflicts between individual patient interests and public interests

The implementation of an epidemic prevention and control system commonly plunges triage nurses into a moral dilemma: they are fearful of being reprimanded for inadequate implementation of epidemic prevention measures, yet the admission of patients infected with COVID-19 may culminate in the occurrence of nosocomial infections. When a triage nurse encounters a critically ill patient with suspected COVID-19, will it threaten the safety of other patients attending the ED if the patient is accepted? If this patient is rejected, the safety of other patients in the ED will be guaranteed, but who will safeguard the life safety of this patient? How should the triage nurse make an ethical choice?

With the intensification of epidemic prevention measures, triage nurses shoulder increased responsibilities and are likely to engage in negative defensive medical behaviors for fear of making mistakes or taking responsibility. A report (30) exhibited that during epidemic prevention and control, a male patient with an angina attack in Xi’an missed the best resuscitation time and died after the Xi’an International Medical Center Hospital refused to admit him on the grounds of epidemic prevention and control because he was from a medium-risk area. What can be reflected through this incident is what should be the relationship between the implementation of the epidemic prevention and control system and the rescue of the lives of patients? The life safety of patients should be given priority, which is the most basic right of patients. Improper implementation of epidemic prevention and control measures and under-triage threaten the most basic right to life of patients.

In addition, ED crowding is probable to exacerbate the spread of the epidemic and likewise endanger the lives of emergency patients. As per the report of the Daily mail, Werribee Mercy Hospital, one of the busiest hospitals in Melbourne of Australia, refused to admit emergency patients due to a shortage of doctors and nurses caused by the impact of the epidemic (31). The ED is overcrowded because of the epidemic. Even if the ED is able to accept patients, the stagnant of patients waiting for treatment at the emergency triage office and the crowded environment can be hindrances to the timely treatment of patients. For patients in extremely critical conditions, the extended triage time can place patients in potentially dangerous situations. Emergency crowding has become a critical factor that threatens the life safety of emergency patients.

### 3.3. Implementation of measures to protect the right to health of the majority: limitations of individual autonomy of patients

The new Code of Ethics for Nurses (2021) points out that nurses need to ensure informed consent for nursing and/or medical care, which includes the right to choose or refuse treatments (24). Restricted autonomy means that the autonomy of patients is not fully respected and used to a certain extent for a wide range of reasons in a particular situation.

During the epidemic prevention and control, medical institution managers introduced a series of epidemic prevention and control measures to protect the safety of medical staff and most patients and for fear of nosocomial infections of COVID-19. In Italy, for example, one of the first epicenters of the COVID-19 pandemic, hospital emergency departments were forced to constantly adapt their space layout to improve safety and comfort for medical staff, including the creation of temporary spaces, buffer zones, “dirty” paths and spaces for medical staff (32). In China, each medical institution set a strict triage system as the first “threshold” for epidemic prevention, the core of which is the requirement for all patients to have a nucleic acid report within 24 or 48 h of presentation to the hospital during the epidemic (33–35). However, dividing the emergency diagnosis and treatment areas of different patients and classifying them based on nucleic acid results may, to some extent, limit the freedom and autonomy of patients. If patients do not hold a negative report, they will need to be tested and receive diagnosis and treatment only after they obtain a negative report. Of course, triage nurses cannot and do not dare to adapt or flexibly use this triage system. In this case, hypothetically, when a patient from a medium-or high-risk area presents to the ED with an acute disease, the triage nurse assesses his or her condition (even though she determines from triage experience that the disease of the patient may not be SARS-CoV-2 infection) and concludes that his or her condition is not life-threatening, and the patient will be required to go to the fever clinic for a nucleic acid test in order to ensure the development of epidemic prevention and can only be admitted to the ED after a negative result is acquired. If patients insist that they are not infected with SARS-CoV-2 and refuse to undergo a nucleic acid test, they will not be admitted to the ED for treatment. Generally speaking, more patients will receive a nucleic acid test due to discomfort with the disease. The autonomy of patients is limited no matter whether patients accept the nucleic acid test or not. In the view of utilitarians, it is appropriate for nurses to choose to sacrifice the autonomy of a few patients for the purpose of implementing the epidemic prevention system and protecting the safety of other patients and staff in the ED. However, in the opinion of deontologists, triage nurses are not motivated by selflessness and altruism, and they are not purely for the sake of patients but altruistic for themselves-they fear reprimand, or they fear not only reprimand but also nosocomial infections. However, in any case, the one-size-fits-all implementation of the epidemic control system brings the autonomy of patients into conflict with the triage decisions of triage nurses.

Asymmetric nurse–patient information can also contribute to the limitation of informed consent and autonomy for patients. During epidemic prevention and control, triage nurses are likely to engage in positive defensive medical behaviors, that is, they include all emergency patients into the scope of potential COVID-19 infection, which is not immoral in terms of their purpose but finally leads to the limited autonomy of patients. Patients, especially patients with lower-level education, may be at a disadvantage when deciding on treatment (36) due to their lack of medical knowledge. Patients are the weaker party in a nurse–patient dyad with asymmetric information, whose informed consent and autonomy are likely to be circumscribed by positive defensive medical behaviors by triage nurses.

### 3.4. Over-triage: waste and “forced” competition of resources for epidemic prevention

Overt-triage often leads to wasted resources for vaccination. The nucleic acid test is taken as an example. The strict triage system and intensive workload may induce overstress of triage nurses during the epidemic, which in turn leads to positive defensive medical triage behaviors. As a result, these behaviors may shift their goal from protecting patients and preventing the COVID-19 infection of patients to preventing nosocomial infections and protecting themselves from leadership reprimands. Abuse of nucleic acid tests caused by over-triage undoubtedly brings about a waste of limited resources for epidemic prevention. Importantly, there are a large number of economically underdeveloped countries in the world that are still unable to conduct universal nucleic acid testing and realize the requirement of ensuring that “all those in need are tested.” Over-triage out of self-preservation is irresponsible in terms of both behaviors and purpose, which does not follow the service concept and principle of “patients’ interests first” for patients and causes a gratuitous waste of restricted epidemic prevention resources for the country.

In the meantime, abuse of nucleic acid tests also give rise to “forced” competition for epidemic prevention resources. That is to say, limited resources for epidemic prevention are meant to serve the patient population that needs nucleic acid tests. Nonetheless, because of over-triage, patients who do not need nucleic acid tests “seize” the finite epidemic prevention resources of those who do need them. This “forced” competition for epidemic prevention resources ultimately results in a “crowding-out” effect, that is, groups in need of nucleic acid tests are crowded out and not effectively tested as a result of the “competition and preemption “for resources.

In addition, there is competition for bed resources among emergency patients. During the emergency phase of the outbreak, there were also challenging decisions to be made by the emergency department staff - new criteria for admission to the emergency intensive care unit (EICU) (37) and the emergency inpatient unit needed to be redefined in the face of bed shortages. When treatment requests from emergency wards and EICU exceed the range of beds, some citizens may not be able to access emergency resources and life-sustaining treatment (37). As a result, emergency medical staff were forced to struggle ethically to choose the patient with the greater success rate of care.

## 4. Directions for improvement in the design and implementation of the triage system: promotion of the ethics of care

EDs in hospitals are in need of a triage system that can comfortably handle major public health events. The triage system should be rebuilt not only to respond to the current epidemic of COVID-19 but also to deal with future public health events that may occur. In the face of a global epidemic, the design of the triage system may not be perfect by considering only the traditional ethical principles of autonomy, beneficence, non-maleficence, and justice (38). On this foundation, we also need to contemplate the deeper ethical issues addressed in the above discussion. In the opinion of the author, there is a need to consider introducing the concept and idea of the ethics of care in the future improvement of the emergency triage system in order to address the ethical issues arising from the current emergency triage system during the epidemic. The ethics of care emphasizes the following points:

Caring is not only a value but also a practice. Specifically, carers should be committed to fulfilling the needs of the cared-for. For medical services, all actions are motivated by caring and cause caring results with the needs of patients as the goal. A triage system that aims at caring and practices caring is a system of kindness and good. Because motivation is the subjective will and the guide to action, the motivation must be good for the development of a triage system. Nevertheless, good motives do not necessarily trigger good actions and do not imply good results. Therefore, the system should be formulated with the consideration that the implementation and the results both are good and kindness. Harm to patients can only be minimized when the epidemic prevention triage system is one that is caring in nature.

### 4.1. Balance of interests between individual patients and patients as a whole in the design of a triage system

Although utilitarians advocate the principle of maximizing interests, there still exists the possibility that maximizing benefits overlooks the interests of a small group of patients. Therefore, the interests and needs of individual patients need to be taken into account while safeguarding the interests and needs of patients as a whole. According to the care ethicist Virgina Held, the ethics of care is suggested to be a sense of moral urgency and to serve and be responsible for meeting the needs of surrounding people, with a primary emphasis on satisfying the needs of dependents (39). She preached that people who are serious about caring for others do not seek primarily to promote their own personal interests and that their interests are intertwined with those of people who they care for (39). In accordance with Held, a pre-assessment of the ethical risks of the implementation of this system must be performed for the design of an emergency triage system that can meet the needs of epidemic prevention and control. In detail, with potential ethical conflicts between the interests of individual patients and patients as a whole presupposed, the system has the responsibility and obligation to create reasonable conditions to reconcile and balance the interests of both parties.

### 4.2. Minimization of conflicts between the implementation of the epidemic prevention system and the rights of patients

In some special situations, it is difficult to predict whether the implementation of a system conflicts with the interests of patients. Moreover, how to ensure that the interests and needs are not infringed? Nel Noddings argues that the one-caring is other-focused and has a motivation shift. The motivation for caring is directed at the well-being and protection of the cared-for. Our focus is on the cared-for, not on ourselves by acting in the interests of the cared-for and with reference to his angle of thinking, objective needs, expectations for us, and the objective components of the problematic dilemma (40). As per this concept of the ethics of care, emergency triage nurses should think about the interests, objective needs, and expectations for medical staff of patients at the moment when conflicts occur between the implementation of the epidemic prevention system and the interests and needs of the emergency patient and consider and settle the problem from his point of view. In conclusion, the interests of patients are ensured not to be infringed upon while maintaining the implementation of a triage system as much as possible with patients at the center of attention and with the premise of not infringing on the interests of others.

### 4.3. “Humanized” design and implementation of triage systems

Nel Noddings believes that caring is primarily about the actions adopted by the one-caring that benefit the cared-for or appear reasonable, with flexibility in these actions, and that the actions of the one-caring are based on the interests of the cared-for without being constrained by rules and regulations (40). The design and implementation of triage systems should not be rigid, and the system should not be designed with “overbear” clauses, nor should the system be implemented. There is a demand to anticipate multiple possibilities and potential ethical issues that may arise in different contexts of triage and to consider the needs and issues of all kinds of patients in a comprehensive manner. When the implementation of epidemic prevention measures may conflict with the life safety of patients, triage nurses should be flexible and adaptable to handle the conflicts, with the ultimate purpose of securing the life safety of patients without harming the interests of others. In addition, in order to ensure the “humanization” of the implementation of emergency triage, the delimitation of triage areas must take into account the psychological health of medical personnel and infected patients, including measures such as optimizing the emergency working environment and triage equipment, and establishing humanized and gentle isolation units, and so on (32).

### 4.4. The design of triage systems should reflect the principles of “actionability” and “empowerment”

Balancing the common objectives of medical institutions and patients is extremely complex. Therefore, in order to bridge the objective gap between both parties and ensure that both parties have the same objective as possible, system design should leave a certain operating space for system implementers. On the one hand, triage systems should be designed in combination with epidemic prevention and control requirements, policy environment, facility and equipment conditions, and other management systems to ensure their actionability. On the other hand, the design of the triage system should take into account the issue of appropriate empowerment of triage nurses in emergency situations (e.g., when patients’ lives are threatened), i.e., providing nurses with certain decision-making authority in triage to increase operability. However, the degree of empowerment in decision-making should be grasped, and the bottom line must be not to harm the interests of others. Triage nurses must make decisions with the life safety of patients as the premise. For instance, if a special event occurs and triage nurses have difficulty in making decisions, they should immediately report to superior managers and make decisions through joint discussion under the premise of ensuring the life safety of patients.

### 4.5. Improvement of triage accuracy and utilization efficiency of epidemic prevention resources

The waste of all resources should be curbed in terms of appropriateness and moderateness in the use of epidemic prevention medical resources. Michael Slote suggests that caring is not only for surrounding people but also for strangers or people in remote areas to some extent (41). The advocacy of the ethics of care can enable hospital managers and triage nurses to become fully aware that they need to care not only for the patient in front of them but also for people in the midst of other epidemics. As a result, they become self-disciplined and more careful in their application of medical resources. The Canadian scholar Jaro Kotalik has reminded us that when discussing the allocation of scarce resources, there is an implicit presupposition that “resource shortages are inevitable,” and unexamined presuppositions can come at the cost of human suffering and loss (42). As far as the utilization of epidemic prevention resources is concerned, the “degree” should be controlled, i.e., the use of epidemic prevention resources should be kept reasonable. On the surface, over-triage only indicates the inappropriate triage behavior of triage nurses, but in essence, involves the reasonableness and legitimacy of the use of epidemic prevention resources. Therefore, improving the accuracy of triage and preventing defensive triage behaviors can, on the one hand, reflect a responsible attitude toward patients and, on the other hand, lay the foundation for elevating the use efficiency of epidemic prevention resources.

## 5. Conclusion

The COVID-19 epidemic raises unique ethical issues about emergency triage, with key issues of patient safety, patient autonomy, and application of epidemic prevention resources. The outbreak and epidemic of COVID-19 provide a crucial opportunity to consider some of the ethical issues exposed by the current emergency triage system. The ethical issues of emergency triage in epidemic situations are unique compared to emergency triage in normalized medical scenarios. The paths to solve these ethical issues can be discussed from the perspectives of system design and implementation with the ethics of care as a theoretical basis, which can provide an idea for the adjustment and the reconfiguration of emergency triage systems in related institutions. Meanwhile, although the discussion of these ethical issues is based on the emergency triage work during the current epidemic prevention and control period, this paper also has key implications for how to improve and refine the emergency triage work in hospitals when major public health events occur in the future.

## Author contributions

LW made a substantial contribution to data analysis and wrote the first draft of the article. XK contributed to the conceptualization, data collecting, and contributed to revising the manuscript. All authors contributed to the article and approved the submitted version.

## Conflict of interest

The authors declare that the research was conducted in the absence of any commercial or financial relationships that could be construed as a potential conflict of interest.

## Publisher’s note

All claims expressed in this article are solely those of the authors and do not necessarily represent those of their affiliated organizations, or those of the publisher, the editors and the reviewers. Any product that may be evaluated in this article, or claim that may be made by its manufacturer, is not guaranteed or endorsed by the publisher.
